# Health system support for childbirth care in Southern Tanzania: results from a health facility census

**DOI:** 10.1186/1756-0500-6-435

**Published:** 2013-10-30

**Authors:** Claudia Hanson, Carine Ronsmans, Suzanne Penfold, Werner Maokola, Fatuma Manzi, Jenny Jaribu, Godfrey Mbaruku, Hassan Mshinda, Marcel Tanner, Joanna Schellenberg

**Affiliations:** 1 Department of Disease Control, London School of Hygiene and Tropical Medicine, London, UK; 2 Department of Infectious Disease Epidemiology, London School of Hygiene and Tropical Medicine, London, UK; 3 Ifakara Health Institute, Dar-es-Salaam, Tanzania; 4 Department of Public Health Science (Global Health), Karolinska Institutet, Stockholm, Sweden; 5 Swiss Tropical and Public Health Institute, Basel, Switzerland; 6 University of Basel, Basel, Switzerland; 7 Tanzania Commission of Science and Technology (COSTECH), Dar-es-Salaam, Tanzania

**Keywords:** Childbirth, Maternal health, Quality of care, Health systems, Access, Tanzania, Sub-Saharan Africa

## Abstract

**Background:**

Progress towards reaching Millennium Development Goals four (child health) and five (maternal health) is lagging behind, particularly in sub-Saharan Africa, despite increasing efforts to scale up high impact interventions. Increasing the proportion of birth attended by a skilled attendant is a main indicator of progress, but not much is known about the quality of childbirth care delivered by these skilled attendants. With a view to reducing maternal mortality through health systems improvement we describe the care routinely offered in childbirth at dispensaries, health centres and hospitals in five districts in rural Southern Tanzania. We use data from a health facility census assessing 159 facilities in five districts in early 2009. A structural and operational assessment was undertaken based on staff reports using a modular questionnaire assessing staffing, work load, equipment and supplies as well as interventions routinely implemented during childbirth.

**Results:**

Health centres and dispensaries attended a median of eight and four deliveries every month respectively. Dispensaries had a median of 2.5 (IQR 2–3) health workers including auxiliary staff instead of the recommended four clinical officer and certified nurses. Only 28% of first-line facilities (dispensaries and health centres) reported offering active management in the third stage of labour (AMTSL). Essential childbirth care comprising eight interventions including AMTSL, infection prevention, partograph use including foetal monitoring and newborn care including early breastfeeding, thermal care at birth and prevention of ophthalmia neonatorum was offered by 5% of dispensaries, 38% of health centres and 50% of hospitals consistently. No first-line facility had provided all signal functions for emergency obstetric complications in the previous six months.

**Conclusions:**

Essential interventions for childbirth care are not routinely implemented in first-line facilities or hospitals. Dispensaries have both low staffing and low caseload which constrains the ability to provide high-quality childbirth care. Improvements in quality of care are essential so that women delivering in facility receive “skilled attendance” and adequate care for common obstetric complications such as post-partum haemorrhage.

## Background

Progress towards reaching Millennium Development Goals four (child health) and five (maternal health) is lagging behind, particularly in sub-Saharan Africa, despite increasing efforts to scale up high impact interventions [1]. Annually 287,000 maternal deaths, between 3.1 to 3.6 million newborn deaths and 2.6 million stillbirths occur globally [2-5]. Most of these deaths take place around birth [4,6].

Having a skilled attendant at birth and a functioning health system – as a part of an enabling environment – backed by a referral level providing emergency obstetric care has been estimated to have the potential to prevent around half of maternal deaths, 27% of stillbirths and 18% of neonatal deaths [7]. Such figures have to be interpreted with caution as evidence is limited [8,9].

The burden of disease resulting from the main causes of maternal and newborn mortality, such as haemorrhage and sepsis, demand prioritization of implementation of key essential interventions such as Active Management of Third Stage of Labour (AMTSL), infection prevention, screening and detection of pre-eclampsia, thermal care of the newborn and immediate and exclusive breastfeeding. These represent highly cost-effective interventions whose importance has been repeatedly confirmed [10-12].

However, not much is known to which extent those interventions such as AMTSL, screening and detection of pre-eclampsia or essential newborn care are fully implemented. Household surveys, which provide coverage data for maternal and child health fail to present information for routine intrapartum care [13]. Health management information system also fail to include indicators of intrapartum care, are often of low quality and incomplete and aggregate data to district or regional level preventing any analysis of care provided at the different levels of care.

In Tanzania, maternal mortality is estimated at 460 deaths per 100,000 live births in 2010 [5]. The most recent DHS in 2010 reported that 96% of women attended ANC at least once and 43% made the recommended four visits in the five years prior the survey. Half (51%) of all births were attended by a health professional and 4.5% of live births were by Caesarean section [14]. In Mtwara and Lindi regions, 41% of women delivered in a health facility in 2006–7. Twenty nine percent of women delivered in a hospital, 2% in health centres and 9% in dispensaries [15].

### The Tanzanian health system

The Tanzanian health system is decentralized to the district level and has a pyramidal structure. Accessibility to primary health care is relative good. Data from Southern Tanzania suggest that about 65% of households were within 5 km to a health facility in 2004 [16].

The dispensaries provide a wide range of basic preventive and curative care including family planning, antenatal care, delivery and postnatal care. Malaria prevention and care including IPTp, screening for syphilis and prevention of mother-to-child transmission (PMTCT) should be available at this level. According to the 2008 Tanzanian “roadmap” for maternal and newborn health (also called ‘One’ Plan) every dispensary should offer basic emergency obstetric and newborn care by 2015 [17]. Dispensaries should be staffed by two clinical officers or assistant medical doctors and two nurses [18].

Health centres are the first referral level and are supposed to offer - in addition to what is available at the dispensary - basic laboratory services and in-patient care. Health centres should have several clinicians, nurses and midwives. The current strategic plan is for half of all health centres to provide comprehensive emergency obstetric care by 2015 [17] to fulfil their role as first referral level. In the past, referral during childbirth was generally directed to hospitals [19,20].

District hospitals, or designated district hospitals (which are run by voluntary agencies such as Missions and contracted by the Ministry of health) offer in-patient and out-patient care and surgical services including Caesarean Sections and blood transfusions [17,21].

The backbone of the Tanzanian health system are clinical officers, who receive 3-year training in general medicine including some training in obstetrics. Clinical officers - or assistant clinical officers who have shorter training - manage the out-patient work in first-line facilities.

Much of the clinical work in hospitals is done by assistant medical officers, a cadre of ‘non-physician clinician’, who first work as clinical officers before they undergo an additional 2-year training including 3-months of obstetric training including obstetric operative care [22,23].

Midwifery is provided by enrolled and registered nurses with 2- and 3-year training respectively. These nurse-midwifery cadres are not entitled to prescribe drugs other than emergency medication [24]. Mother and child health aides (MCHA) have 2-year training in mother and child health care and are counted as skilled attendants. They are being gradually replaced by nurses. Nursing assistants receive 1-year training in nursing and are not considered to be skilled childbirth attendants.

### Strategies in maternity care

Reproductive and child health services including vaccination and family planning are free of charge in public health facilities, but indirect costs (transport and supplies) and unofficial payments reportedly occur [25,26]. A four-visit focused ANC strategy was introduced in 2002, emphasising malaria prevention and syphilis screening, early detection of danger signs as well as promotion of health facility delivery [27,28].

Specific training in emergency obstetric care was introduced in 2001. Improvements in maternal health services are supported by various partners and the “ONE” strategy of UN partners [29]. At district level maternal and newborn health is funded through the “basket fund” mechanism where joint donor financing of $ US 1 per capita (2011) is made available [30-32]. Funds from central government, (district) council’s own resources and health insurance schemes are also available to the council health management team for employment, training, drugs and equipment.

Much information from Tanzania is available on the quality of care and interventions provided during antenatal care from national surveys [14,33] and research studies [28,34-36]. There are several studies at national and sub-national level on the availability of emergency obstetric care (EmOC) focusing on seven signal functions [37]. These studies reported that basic EmOC is only available in a small minority of first-line health facilities (dispensaries and health centres) [38-40].

Relatively few reports from Tanzania focus on the quality of care provided for routine childbirth. Data is lacking on the extent of implementation of key interventions to reduce the burden of maternal and newborn ill-health such as AMTSL or foetal monitoring. Such information is important to strategize improvement approaches for better quality. Further, the best balance between availability, accessibility and quality can only be achieved if the optimal mix of interventions is based on health system characteristics in specific settings.

The aim of this study is to describe routine care offered during childbirth at dispensaries, health centres and hospitals in rural Southern Tanzania. We used data collected during a health facility census in five districts in 2009. By combining health facility data with the proportion of institutional births from population-based data, we estimate the percentage of births receiving an essential delivery care package. Against the background of relative good access to health care in we briefly discuss implications of results for organisation of childbirth care services.

## Methods

### Study area

This study is part of an investigation into the epidemiology of maternal mortality undertaken in five districts (Lindi Rural, Nachingwea, Ruangwa, Newala and Tandahimba districts) in Lindi and Mtwara regions in Southern Tanzania. The total population was 890,939 in 2002 [41] and there were 22,243 live births in 2007 [15]. The districts are predominantly rural and people live from subsistence farming, fishing and small scale trading. Most roads are unpaved and often impassable during the rainy season. The median distance to the nearest health facility was 3.2 km with an interquartile range from 0.8 km to 5.2 km.

### Study basis and methodology

The study was undertaken within the framework of the ongoing ‘Improving Newborn Survival in Southern Tanzania’ (INSIST) randomised controlled trial (clinical trial number NCT01022788) and aimed at generating baseline information on the structure and function of health services for maternal and newborn care in five districts in Southern Tanzania.

The study tool was adapted from publicly available tools including the Safe Motherhood Needs Assessment and the EmOC monitoring tool [37,42]. A modular check-list type questionnaire was used. The first module, directed to the head of the health facility, assessed services routinely offered, as well as staff employed and training received for first-line facilities. The second module on equipment and supplies included an assessment of presence and functionality by the survey team. A third reviewed the health facility records for the year 2008 and abstracted information on workload. The fourth module was directed to all staff working in the reproductive health clinic and assessed implementation levels of essential interventions recommended as part of essential childbirth care [11,43]. A five-answer option ranging from “always implemented” to “never done” was used to get information on functionality of childbirth and implementation routines. This assessment methodology was meant to substitute direct observations of deliveries which could not be done due to the low caseload in first-line facilities. The information on implementation levels were summarised into an essential childbirth care package defined based on standard publications from WHO [43,44] and included AMTSL, partograph use including foetal monitoring, infection prevention, breastfeeding promotion, thermal care and prevention of ophthalmia neonatorum. A weighted analysis was performed to reflect the distribution of care seeking by mothers (share of deliveries in hospitals, health centres and dispensaries) to calculate the proportion of mothers having received essential childbirth care. This measurement was applied to the proportion of institutional delivery of 41% reported for 2007 study in the area [15] to compute a population figure for women having received “essential childbirth care”. Emergency obstetric care was assessed asking when it was the last time the health facility had encountered selected obstetric complications and implemented any of the interventions known as signal function as part of the basic EmOC monitoring approach [37].

The questionnaires were administered in Swahili.

Data was collected in March 2009 by trained interviewers. Pairs of interviewers visited every facility without prior notice. Revisits were not undertaken if the facility was closed. Data quality management included daily review of collected data by a supervisor with regular feedback and a repetition of a subset of questions in selected health facilities.

### Data processing and analysis

Personal Digital Assistants were used for data collection. A modular questionnaire was developed using Pendragon Forms 4.0 software. Logical checks and skip patterns were performed. Information was downloaded daily onto laptop computers and backups made. Daily summary reports were produced to ensure completeness of data collection.

Data analysis was performed using STATA 11 [45]. We tabulated frequency of availability of services and equipment for dispensaries, health centres and hospitals. Chi-squared tests were carried out to assess the association between availability of services and supplies and levels of care.

### Ethical approvals

Ethical approval was received from the local and national institutional review boards (Ifakara Health Institute and the National Tanzania Medical Research Co-coordinating Committee, through the Tanzania Commission for Science and Technology) and from London School of Hygiene and Tropical Medicine, UK.

Prior to visiting the health facility written consent to approach the facilities to participate in the study was obtained from each Council Health Management Teams and a copy of the permission letter given to each facility before data collection was started.

## Results

A total of 163 health facilities were identified in the five districts. Of these, data could not be obtained from three facilities due to unavailability of staff. One private-for-profit health centre was excluded because it did not provide care to mothers and children. Health facilities with information included 6 hospitals, 13 health centres and 140 dispensaries. Two hospitals, one health centre and three dispensaries were private-non-profit (Mission) facilities, one was a private-for-profit and all others were public facilities.

**Figure 1:**
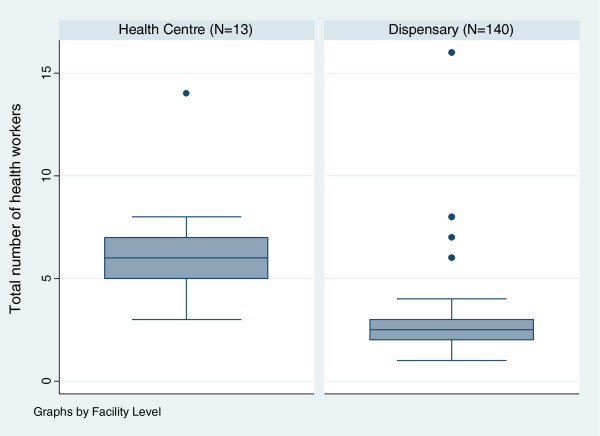
**Box Plot showing the number of health providers employed at first-line health facilities, for health centres and dispensaries.** (Boxes represent the data within the interquartile range (IQR) from the 25^th^ to 75^th^ percentile. The lines represent the range of the data (minus outliers that are data points that lay more than 1.5 fold above or below the IQR).

**Figure 2:**
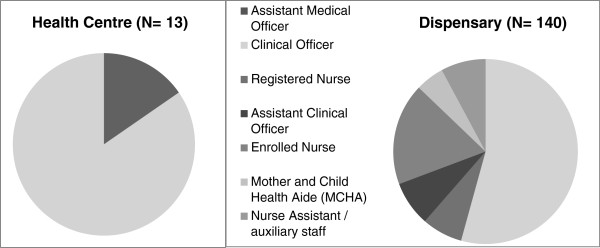
Staff category in-charge of health centres and dispensaries.

Staffing levels and qualifications are shown in Figures 1 and 2. A median of six (interquartile range [IQR] 5–7) and 2.5 (IQR 2–3) health workers were employed at health centres and dispensaries, respectively. An assistant medical officer was in-charge of two (15%) health centres and the remaining 11 health centres were headed by a clinical officer. Seventy six (54%) dispensaries were headed by clinical officers. In 18 dispensaries (13%) a MCHA or nurse assistant was in-charge of the dispensary.

Two health centres (15%) and 64 dispensaries (46%) had no certified midwifery staff (registered nurse or enrolled nurse)(data not shown). Out of the 64 dispensaries without any staff with certified midwifery skills, 18 dispensaries (13%) had at least one MCHA, but 41 (29%) only had a nurse assistant in-charge of reproductive health services.

**Figure 3:**
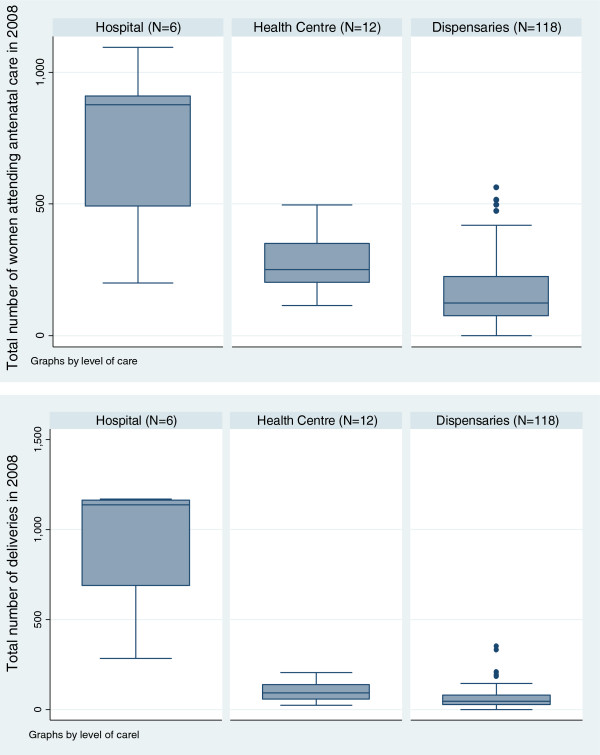
Box plots showing the median number of women seen for antenatal care (first visit) and delivery care in the year 2008 (12 months period) for hospitals, health centres and dispensaries.

Antenatal and delivery care was offered in all six hospitals and 13 health centres. One hundred thirty-five (96%) dispensaries offered ANC and 131 (94%) provided delivery services. Data on workload was not available for one health centre and 13 dispensaries. The hospitals, health centres and dispensaries saw a median of 878 (IQR 491–910), 251 (IQR 200–350) and 125 (IQR 75–225) pregnant women respectively for ANC (first visit) in 2008. The respective figures for the number of deliveries were higher for the hospitals (median 1137, IQR 689–1163) but lower for health centres (median 92, IQR 57–140) and dispensaries (median 48, IQR 26–81). Thus 5579 (37%), 1320 (9%), 8118 (54%) of the reported institutional deliveries in 2008 were in hospitals, health centres and dispensaries, respectively (Figure 3).

### The quality of ANC and childbirth care

Tetanus vaccination, counselling for family planning, bed nets and birth preparation was almost universally available (see Table 1). Blood pressure measurement was not routine in one hospital, one health centre and 33 (28%) dispensaries and a urine protein test for pre-eclampsia diagnosis was only performed in 6 (55%) health centres and five (4%) dispensaries.

**Table 1:** Availability of antenatal and essential delivery care for mother and newborn (self reports) and essential supplies and equipment by hospital, health centre and dispensary

	**Hospital**	**Health centre**	**Dispensary**	**Chi-squared p-value^**
	**%**	**%**	**%**	
**Interventions and services offered during antenatal care**				
	N = 6	N = 11	N = 119	
Screening and preventive intervention				
Tetanus vaccination offered	100	100	97	0.755
IPTp offered	100	91	94	0.748
Blood pressure measurement offered	83	91	72	0.350
Urine protein test offered	100	55	4	<0.001
Haemoglobin test offered	100	55	19	<0.001
PMTCT offered	100	100	79	0.112
Syphilis testing offered	100	100	43	<0.001
Counselling				
Family planning counselling	83	100	98	0.045
Bed net/voucher promotion	100	100	94	0.590
Birth preparation counselling	100	100	99	0.931
Danger sign counselling	100	100	100	-
**Essential delivery and newborn care**				
	N = 6	N = 13	N = 131	
Injectable uterotonics as part of AMTSL always injected	100	77	57	0.045
Cord traction/massage as part of AMTSL always done	100	69	59	0.106
Partograph always used	100	85	63	0.063
Fetal heart beat always recorded	100	100	82	0.139
Blood pressured always measured	67	69	64	0.930
Infection prevention measures always used	100	92	94	0.798
Encouragement of breastfeeding always done	100	100	95	0.587
Wrapping/drying of baby always done	100	100	95	0.636
Application of eye ointment always done	83	92	50	0.006
**Available equipment and supplies**				
	N = 6	N = 13	N = 129	
Sulphadoxine-Pyrimethamine (for IPTp)	100	92	86	0.507
HIV tests	100	92	82	0.350
Syphilis tests	67	85	43	0.008
Uterotonics	100	100	69	0.017
Functioning blood pressure apparatus	83	100	55	0.003
Functioning means of sterilisation	83	100	88	0.381

^p-value for difference between level of care.

PMTCT was offered in 94 (79%) dispensaries whereas syphilis screening was only available in 51 (43%) dispensaries.

Injection of uterotonics, cord traction and uterus massage as part of AMTSL was implemented in all hospitals, nine (69%) health centres and 37 (28%) dispensaries. Uterotonics were available in all hospitals and health centres and 90 (69%) dispensaries.

All hospitals, 11 (85%) health centres and 83 (63%) dispensaries stated that they always monitor labour with help of a partograph. However two hospitals (33%), four health centres (31%) and 47 dispensaries (36%) reported that they do not monitor blood pressure regularly as part of delivery care. Functioning blood pressure meters were available in five (83%), 13 (100%) and 72 (55%) of hospitals, health centres and dispensaries respectively.

**Figure 4:**
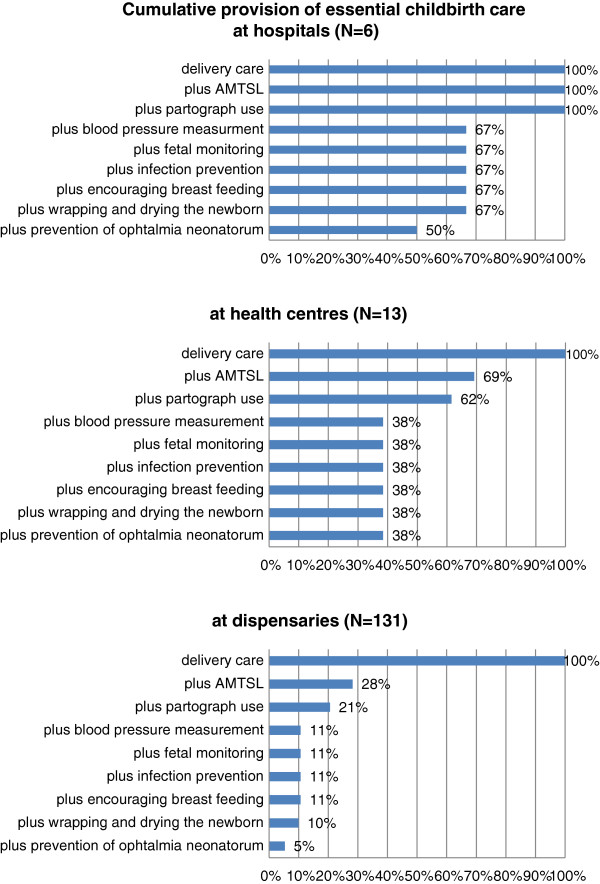
Cumulative provision of essential interventions for mothers and newborns during delivery by hospitals, health centres and dispensaries.

Only three hospitals (50%), five health centres (38%) and seven dispensaries (5%) can be rated as providing a minimum package of ‘essential childbirth care’ using the selected eight essential interventions for childbirth care (see Figure 4). The weighted analysis based on the number of deliveries in 2008 in dispensaries, health centres and hospitals, suggested that only 25% of institutional births or 10% of all women who gave birth in 2008 in the study area received an essential childbirth care package.

No hospital or first-line health facility reported having seen all of the five major obstetric complications during the six months prior to the survey, thus no facility qualified as providing basic EmOC. The most common complications were post-partum haemorrhage and obstructed labour (see Table 2). The most frequent obstetric interventions were manual removal of the placenta and neonatal resuscitation. Fourteen (11%) and 22 (17%) dispensaries reported having performed these two interventions at least once during the past six months. Assisted delivery was rare, reported by only one hospital and two health centres.

**Table 2:** Major obstetric complication, emergency obstetric intervention and equipment by hospitals, health centres and dispensaries (proportion of health facilities reporting having seen the complication or having performed the intervention at least once during the past 6 months prior the survey)

	**Hospital**	**Health Centres**	**Dispensary**	**Chi-squared p-value^**
	**%**	**%**	**%**	
	**(N = 6)**	**(N = 13)**	**(N = 131)**	
**Major obstetric complications**				
Post-partum haemorrhage	50	31	21	0.436
Obstructed labour	83	77	28	<0.001
Puerperal Sepsis^#^	17	23	2	<0.001
Eclampsia	50	0	3	<0.001
Complications from incomplete/unsafe abortion	67	39	13	0.002
**Signal functions**				
Manual removal of the placenta	50	23	11	0.058
Assisted delivery	17	15	4	<0.001
Parenteral sedatives given	33	0	1	<0.001
Parenteral antibiotics for puerperal sepsis^#^	100	8	5	<0.001
Removal of retained abortion residuals	50	31	1	<0.001
Newborn resuscitation	83	15	17	0.002
**Equipment and supplies** (functioning and available at day of visit)				
Vacuum extractor (assisted delivery)	67	15	2	<0.001
Magnesium Sulfate	83	62	57	0.413
iv. Antibiotics (i.v ampicillin, i.v. & oral metronidazole & i.v. gentamycine)^$^	100	23	8	<0.001
Amoxicillin/Ampicilline & metronidazole, both orally*	50	7	3	<0.001
Manual Vacuum Aspiration (MVA)	100	46	0	<0.001
Newborn ambu bag	100	69	31	<0.001

^p-value for difference between level of care.

^#^Inconsistent reporting.

^$^WHO recommendation *standard for moderate severe puerperal sepsis in Tanzania.

## Discussion

There are three major challenges for childbirth care in rural Southern Tanzania. First, dispensaries are inadequately staffed to provide quality childbirth care on a 24 h/7d basis. Secondly, coverage levels for essential childbirth care interventions such as AMTSL, screening for pre-eclampsia and infection prevention measures including prophylaxis of ophthalmia neonatorum are insufficient even at hospital level. Thirdly, the number of deliveries in dispensaries and health centres is low.

Staffing levels were well below national standards for clinicians and midwifery staff particularly at dispensary level as described elsewhere [16,33,46,47]. Compared to studies investigating into staffing in the 1990s our data suggest that MCHA and assistant clinical officers, have been to some extent replaced by better qualified staff [48,49]. But despite this, half of dispensaries still did not have certified midwifery staff in 2009.

The low caseload in dispensaries and health centre is likely due to several factors as other studies have indicated such as preference for home deliveries [50], low perceived quality of care [51] a preference to deliver in faith-based facilities or hospitals despite higher costs [52,53], and the density of health facilities. Low caseload may compromise the technical quality of care [54]. Although no threshold of minimum caseload has been put forward, it has been suggested that midwives might handle up to 175 deliveries per year [55] which allows them to experience and regularly handle complications such as postpartum haemorrhage. The fact that only 2% of dispensaries reported a case of eclampsia and 1% had given sedatives in the past six months further supports that in this setting either skill maintenance has to be ensured through strong supervision and regular obstetric drills or delivery care needs to be more centralised [56].

Health centres seem to be greatly underused, despite our data suggesting that the quality of care was substantially better (38% providing all selected essential interventions compared to 5% of dispensaries). Factors contributing to low use of health centres might be that their role in delivery care is ill-defined [19,20]. Perceived quality of care might be low partly also as women might not be sufficiently informed about the better technical quality at health centres. Perceived quality of care is a major driver and hence many studies suggest that women prefer to deliver at higher level facilities despite increased distance and costs [52,57,58].

The health facility census echoes the known deficiencies with regard to critical interventions. The levels of critical interventions were broadly similar to levels reported by the Tanzania Service Provision Assessment [33] and the latest DHS [14]. Coverage was high for interventions such as vaccination and prevention of malaria and HIV, where global initiatives support implementation. Low coverage levels were found for measurement of blood pressure, haemoglobin and urine protein as also reported elsewhere [36,59,60].

The low implementation level of the very cost effective and technically easy interventions such as AMTSL and blood pressure screening for pre-eclampsia during pregnancy and childbirth at all levels of care were striking. The low adequate implementation of AMTSL has also been reported previously from Tanzania [61]. The data suggest that the low implementation can not to be entirely explained by the lack of uterotonics. Health workers often explain ‘saving’ the oxytocics for cases of postpartum haemorrhage (data not shown).

Other major deficiencies were seen in availability of sterilization equipment. Providers often have to use the second or third best option for sterilization and might put patients at risk as described in a quality assessment which was complementing this health facility census [62].

Partographs have been reported to be used in hospitals in Tanzania, but not always in a satisfactory manner [63-65]. We reported relative high proportion of general usage but insufficient recoding of foetal and maternal wellbeing. Low use of assisted deliveries has also been reported from referral institutions in Tanzania with levels around 2% of deliveries [66,67].

The described deficits in provision of essential childbirth care questions that the national strategy of upgrading all dispensaries to provide basic EmOC by 2015 can be achieved [17]. Although there are improvements over the past 10 years the human resource gap is still wide. Limited training capacities [46] and general budget ceilings limit the expansion of health staff in the government sector.

### Limitations

Our study was primarily a structural assessment based on reports of staff and observations of availability of commodities, but neither included observations of ANC visits or delivery care, nor exit interviews. Thus demand side factors, provider-client interactions and client perceived quality of care which are important direct functional components were not part of this study.

Further, the reported levels of care are likely to be biased by social desirability and may give an overly positive view of the quality of childbirth care. The effect might be greater for reports on counselling activities than for investigations, screening or services as also described in another study in Tanzania [28].

## Conclusions

Our study indicates low quality of care and low utilization of childbirth care services at first-line facilities, and major deficiencies in the availability of human resources, commodities for childbirth care and implementation of essential interventions at all levels. The low caseload in dispensaries and health centres constrains skills maintenance, as many complications are unlikely to be seen more than once a year. The relative good geographical accessibility of facilities in southern Tanzania is constraint by the low availability of essential services pointing to the tension between prioritizing quality of care and accessibility in recourse poor settings. Prioritizing provision of highest-impact interventions such as AMTSL, screening and referral for pre-eclampsia, and care for most frequent complications such as postpartum haemorrhage and retained placenta might be a way forward in view of the resource constraints.

## Abbreviations

AMTSL: Active management of third stage of labour; ANC: Antenatal care; EmOC: Emergency obstetric care; IPTp: Intermittent preventive treatment for malaria during pregnancy; IQR: interquartile range; MCHA: Mother and child health aides; PMTCT: Prevention of mother-to-child transmission.

## Competing interests

The authors have declared that they have no competing interests.

## Authors’ contributions

CH was involved in design, analysis and interpretation of the study, and writing of the manuscript. CR and GM supported the analysis and helped to write the manuscript. SP, WM, FM, HS & MT were involved in the conception, design and implementation of the field work. JS was involved in design, implementation, and interpretation of the study and supported the writing of the manuscript. All authors contributed to the revision of the manuscript and read and approved the final manuscript.
